# Determinants of Vitamin D Status of Women of Reproductive Age in Dhaka, Bangladesh: Insights from Husband–Wife Comparisons

**DOI:** 10.1093/cdn/nzz112

**Published:** 2019-10-07

**Authors:** Joo-Hyun Jeong, Jill Korsiak, Eszter Papp, Joy Shi, Alison D Gernand, Abdullah Al Mahmud, Daniel E Roth

**Affiliations:** 1 Department of Nutritional Sciences, University of Toronto, Toronto, ON, Canada; 2 Centre for Global Child Health, Hospital for Sick Children, Toronto, ON, Canada; 3 Department of Nutritional Sciences, The Pennsylvania State University, University Park, PA, USA; 4 Centre for Child and Adolescent Health, International Centre for Diarrhoeal Disease Research, Bangladesh, Dhaka, Bangladesh; 5 Department of Paediatrics, Hospital for Sick Children & University of Toronto, Toronto, ON, Canada

**Keywords:** vitamin D, Bangladesh, women, pregnancy, spouses

## Abstract

**Background:**

Vitamin D deficiency is common among women of reproductive age (WRA) in Bangladesh, but the causes remain unclear.

**Objective:**

To explain the high prevalence of vitamin D deficiency in WRA in Dhaka, Bangladesh, we compared the vitamin D status of pregnant women with that of their husbands and between pregnant and nonpregnant states.

**Methods:**

This study was an observational substudy of the Maternal Vitamin D for Infant Growth trial conducted in Dhaka, Bangladesh. Women (*n* = 1300) were enrolled in the second trimester of pregnancy and randomly assigned to 1 of 5 arms consisting of different doses of vitamin D supplements or placebo, with 1 arm continuing supplementation until 6 mo postpartum. A subgroup of trial participants and their husbands with plasma 25-hydroxyvitamin D [25(OH)D] concentration measurements (*n* = 84), and placebo-group trial participants with serum 25(OH)D measured in the second trimester of pregnancy and 6 mo postpartum (*n* = 89) were studied using linear mixed-effects regression models.

**Results:**

The mean ± SD plasma 25(OH)D in pregnant women in the second trimester was 23 ± 11 nmol/L. Adjusting for age and season, 25(OH)D of pregnant women was 30 nmol/L lower (95% CI: −36, −25 nmol/L) than that of men. Only 9% of total variance in 25(OH)D was explained by factors shared by spousal pairs. Selected nonshared factors (BMI, time spent outdoors, involvement in an outdoor job, sunscreen use) did not explain the association of sex with 25(OH)D. Adjusting for age, season, and BMI, 25(OH)D was similar during pregnancy and 6 mo postpartum (mean difference: −2.4 nmol/L; 95% CI: −5.3, 0.4 nmol/L).

**Conclusions:**

In Dhaka, WRA have substantially poorer vitamin D status than men. Variation in 25(OH)D is not greatly influenced by determinants shared by spouses. Measured nonshared characteristics or pregnancy did not account for the gender differential in 25(OH)D. This trial was registered at clinicaltrials.gov as NCT01924013.

## Introduction

Vitamin D has an essential role in calcium and phosphorus homeostasis, skeletal growth, and bone health (1). Some evidence suggests that vitamin D status may influence the risks of acute and chronic diseases, including musculoskeletal disorders, cancer, cardiovascular diseases, autoimmune diseases, infections, and diabetes mellitus (2). Humans acquire vitamin D by cutaneous synthesis upon ultraviolet B (UVB) radiation and, usually to a lesser extent, through diet (e.g., fatty fish, eggs, and fortified products) or supplements (1). Biological factors such as older age and obesity can limit the synthesis and bioavailability of vitamin D (3). The acquired

vitamin D is converted to 25-hydroxyvitamin D [25(OH)D], the main circulating metabolite of vitamin D and an accepted marker of an individual's vitamin D status (4).

Environmental factors such as latitude and season determine vitamin D status by modifying UVB exposure (3, 5, 6). However, numerous studies have shown that vitamin D deficiency is prevalent in many countries where abundant UVB exposure from sunlight is expected (7–9). Women may be particularly at risk owing to behaviors that limit sun exposure such as conservative clothing practices based on cultural norms, liberal use of sunscreen, and indoor lifestyles (10–12). Studies in Bangladesh have revealed a high prevalence of vitamin D deficiency in women of reproductive age (WRA) (11, 13, 14). In a randomized trial designed to evaluate the dose-dependent effects of prenatal and postpartum vitamin D supplementation on infant length in Dhaka, Bangladesh (15), the majority of women in the second trimester of pregnancy had serum 25(OH)D concentrations  < 30 nmol/L, a conventional cutoff for vitamin D deficiency (1).

Vitamin D deficiency in WRA may have public health importance because of its established influence on neonatal vitamin D stores and hypothesized effects on birth outcomes (16–19). However, identifying and understanding the factors that contribute to vitamin D deficiency in WRA are required to develop and implement feasible preventative strategies for at-risk populations. Comparison of the vitamin D status of spousal (i.e., husband and wife) pairs enables the distinction of the overall effect on vitamin D status of factors that would be typically shared by spouses living within the same household (e.g., availability of vitamin D–rich foods, socioeconomic status) from individual-level factors that would not be expected to be shared by spousal pairs (e.g., UVB skin exposure, biological differences in vitamin D metabolism between men and women). Even in the absence of direct measurements of such factors, husband–wife comparisons enable the partitioning of the observed variance in 25(OH)D in a particular setting into within-pair and between-pair components, thereby yielding an understanding of the type of factors that contribute to low 25(OH)D concentrations among WRA.

In this substudy of a randomized trial cohort in Dhaka, Bangladesh, we aimed to *1*) compare 25(OH)D concentrations of pregnant women and their husbands and estimate the correlation of 25(OH)D concentrations within spousal (husband and wife) pairs; *2*) determine if selected nonshared factors (BMI, time spent outdoors, involvement in an outdoor profession, and sunscreen use) explain the association between sex and 25(OH)D concentrations; and *3*) compare 25(OH)D concentrations at the second trimester of pregnancy with 6 mo postpartum in Bangladeshi women and estimate the within-woman correlation of 25(OH)D concentrations at these 2 time points. Whereas the first 2 aims address the determinants of husband–wife differentials in vitamin D status, the third aim addresses the potential effect of pregnancy on 25(OH)D, primarily to ensure that comparisons of 25(OH)D concentrations between pregnant women and their spouses provided a meaningful comparison of WRA with men.

## Methods

This was an observational substudy of the Maternal Vitamin D for Infant Growth (MDIG) trial (NCT01924013) conducted in Dhaka, Bangladesh (24°N) (15, 19). Women (*n* = 1300) were enrolled in the second trimester of pregnancy (17–24 weeks of gestation) and randomly assigned to 1 of 5 arms consisting of different doses of vitamin D supplements or placebo, with 1 arm continuing supplementation until 6 mo postpartum. Women's venous blood specimens, anthropometric measurements, and questionnaires were collected at scheduled intervals until 6 mo postpartum, whereas each husband's data and blood specimens were collected once, as soon as possible after their wife's enrollment. Specimen collection occurred between March 2014 and July 2016. The MDIG trial protocol was approved by the research ethics committees at The Hospital for Sick Children (Toronto, Canada) and the International Centre for Diarrheal Disease Research, Bangladesh (icddr,b; Dhaka, Bangladesh). All participants were enrolled after providing written informed consent at the Maternal and Child Health Training Institute, a Bangladeshi national government–operated facility in central Dhaka. Participants were compensated for costs of transportation and time away from home and/or work. Detailed methods of the trial were described previously (19).

### Data sources

25(OH)D measurements were available from 84 spousal pairs, who were among the first to enroll in the MDIG trial. Height and weight were measured at the time of specimen collection among men (close to the time of their wife's enrollment). For women, the blood draw was conducted at baseline (second trimester of pregnancy) before vitamin D/placebo supplementation was started, and height and weight at that time were used to calculate baseline BMI (in kg/m^2^). The trial excluded participants who were prescribed vitamin D supplements by their physician or were unwilling to stop taking nonstudy vitamin D or other supplements containing vitamin D or calcium (∼16% of women who underwent detailed screening for enrollment were excluded for these reasons). Other hypothesized mediators (time spent outdoors, involvement in an outdoor job, and sunscreen use) were characteristics selected from self-reported questionnaires administered to both men and women at the time of specimen collection. A separate group of women in the placebo group (i.e., not supplemented with any vitamin D) who had 25(OH)D measured at both baseline and 6 mo postpartum were included in analyses of the effect of pregnancy on 25(OH)D concentrations (*n* = 89). Maternal age (in years) was only collected at enrollment; therefore, age at 6 mo postpartum was estimated by adding 1 y to the second-trimester age. The height and weight for this cohort of women were also measured per standard protocol at trial enrollment (second trimester of pregnancy) to calculate pregnant BMI and at 1 y postpartum to estimate nonpregnant BMI. Only 5 women were included among both the 84 spousal pairs and the 89 women from the placebo group. Vitamin D status was determined by measuring plasma or serum 25(OH)D_3_ concentrations using HPLC tandem MS at the Analytical Facility for Bioactive Molecules laboratory, The Hospital for Sick Children, Toronto, Canada (15). Only plasma samples were available for men; therefore, the spousal comparisons were based on plasma 25(OH)D, whereas the comparison of pregnancy with postpartum was based on serum 25(OH)D. This approach was justified by a separate analysis of women participants from the trial who had both serum and plasma 25(OH)D measured in the same blood specimens; there was a strong positive correlation between the plasma and serum 25(OH)D measurements (*r* = 0.9, *n* = 107, *P* < 0.001) but the median of plasma concentrations (18 nmol/L) was slightly lower than that of the serum measurements (23 nmol/L). 25(OH)D_2_ concentrations were undetectable or negligible in all samples tested, and thus excluded from analyses. The C3 epimer of 25(OH)D_3_ was also excluded.

### Statistical analysis

Baseline characteristics of husbands and wives were compared using paired *t* tests for normally distributed continuous variables, Wilcoxon’s Signed Rank tests for continuous variables that were not normally distributed, McNemar tests for dichotomous variables, and the Marginal Homogeneity test for categorical variables (with >2 categories). Similar tests were used to compare characteristics of women from the placebo arm during and after pregnancy. Average 25(OH)D concentration in each group was expressed as mean ± SD.

To address aim 1, a linear mixed-effects regression model with a random intercept was used to estimate the association between sex and 25(OH)D concentration, and the within-pair correlation was the model-derived intraclass correlation coefficient (ICC). To assess the role of mediators in the association between husband and wife vitamin D status (aim 2), separate models were run in which the hypothesized mediator was added to the linear mixed-effects regression model, and the coefficient for sex was compared between when the variable was added to the model and when it was excluded from the model. The models were repeated without sex as a covariate to test each mediator's separate effect on 25(OH)D. To address aim 3, a linear mixed-effects regression model with a random intercept was used to determine the association between pregnancy and 25(OH)D concentrations, and the within-woman correlation was the ICC.

Models for aims 1 and 2 were adjusted for age and season of specimen collection because these factors can influence the cutaneous synthesis of 25(OH)D. BMI was not adjusted for in this model because this factor was tested as a potential mediator in aim 2. The model for aim 3 was adjusted for age, season, and BMI. Age (in years) and BMI were included as continuous variables, whereas season of specimen collection was categorized as March–May, June–August, September–November, and December–February. Data analysis was performed using STATA version 14 software (StataCorp LLC).

## Results

Compared with pregnant women, husbands were significantly older, had lower BMI, spent more time outdoors, were more likely to be employed in outdoor occupations, and were less likely to use sunscreen regularly (**Table 1**). Specimen collection in women occurred predominantly from March to May, whereas men's samples were collected from March to August. Women's specimens were collected a median of 29 d before their husbands’.

**TABLE 1:** Characteristics of men and women from spousal pairs and women compared by time point during and after pregnancy in Dhaka, Bangladesh^1^

Characteristic	Men (*n* = 84)	Women (*n* = 84)	*P* ^2^	Women at second trimester of pregnancy (*n* = 89)	Women at 6 mo postpartum (*n* = 89)^3^	*P* ^4^
Age,^5^ y	30.5 [27.0–34.0]	22.0 [19.0–26.0]	<0.001	22.0 [20.0–25.0]	23.0 [21.0–26.0]	—
BMI,^6^ kg/m^2^	22.2 [19.9– 24.5]	22.9 [20.9–25.2]	0.038	23.5 [20.7–26.9]	22.9 [19.9–26.7]	0.927
Month of blood collection						
March–May	49 (58.3)	76 (90.5)	<0.001	40 (44.9)	25 (28.1)	0.023
June–August	35 (41.7)	8 (9.5)		15 (16.9)	13 (14.6)	
September–November	0 (0.0)	0 (0.0)		12 (13.5)	15 (16.9)	
December–February	0 (0.0)	0 (0.0)		22 (24.7)	36 (40.5)	
Time spent outdoors per day,^7^ h	2.0 [1.0–3.0]	1.0 [1.0–1.0]	0.002	1.0 [1.0–1.0]		
Engaged in an outdoor occupation	21 (25.0)	0 (0.0)	<0.001	0 (0.0)		
Sometimes or always used sunscreen^8^	1 (1.2)	14 (16.9)	<0.001	4 (4.5)		

1 Values are *n* (%) or median [IQR] unless otherwise indicated.

2 Comparison of values between men and women using paired *t* tests for BMI, Wilcoxon's Signed Rank tests for age and “time spent outdoors,” McNemar tests for “engaged in an outdoor occupation” and “sometimes or always used sunscreen,” and the Marginal Homogeneity test for month of blood collection.

3 The potential mediators (time spent outdoors, engaged in an outdoor occupation, and sunscreen use) were not measured at this time point.

4 Comparison of values of women during their second trimester of pregnancy and at 6 mo postpartum using paired *t* tests for BMI and the Marginal Homogeneity test for month of blood collection.

5 Age of women at 6 mo postpartum was estimated by adding 1 y to the age reported at enrollment in the second trimester of pregnancy; therefore, no statistical test for the difference between time points is reported.

6 Postpartum BMI based on weight and height measured at 1 y postpartum; owing to missing data, *n* = 86 at the postpartum time point.

7 Owing to missing data, *n* = 83 women included in the comparison of men with women and *n* = 88 women at the second trimester of pregnancy (in the comparison of the second trimester of pregnancy with 6-mo postpartum).

8 Owing to missing data, *n* = 83 men and *n* = 83 women (in the comparison of men with women).

**FIGURE 1:**
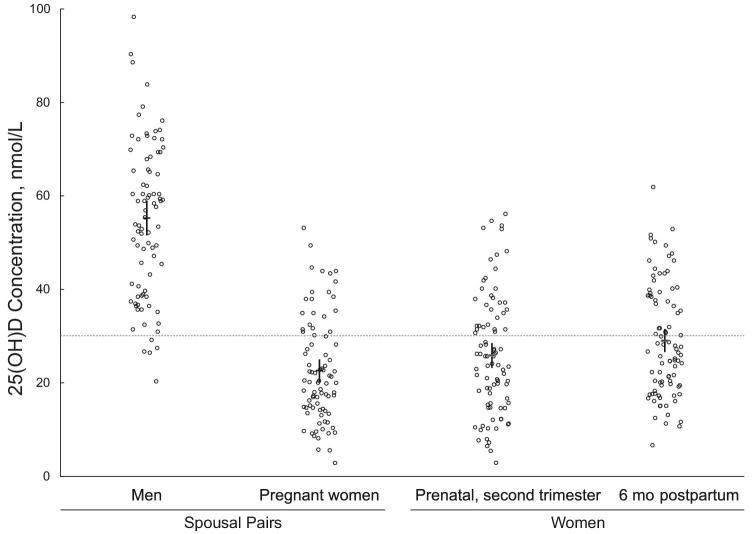
Distributions of plasma 25(OH)D concentrations of pregnant women (second trimester) and their husbands (*n* = 84 pairs), and serum 25(OH)D concentrations of women during pregnancy (second trimester) and 6 mo postpartum (*n* = 89). Group means are shown as horizontal lines, whereas vertical lines represent the 95% CIs. Among spousal pairs, the mean plasma 25(OH)D concentration for men (55 nmol/L; 95% CI: 51, 59 nmol/L) was significantly higher than that of women (23 nmol/L; 95% CI: 20, 25 nmol/L; *P* < 0.001 for the comparison of men with women). The mean serum 25(OH)D concentration at pregnancy (26 nmol/L; 95% CI: 23, 29 nmol/L) was lower than at the postpartum measurement (29 nmol/L; 95% CI: 27, 32 nmol/L; *P* = 0.03 for the comparison of pregnancy and postpartum). The dotted horizontal line indicates the conventional cutoff for vitamin D deficiency (30 nmol/L). 25(OH)D, 25-hydroxyvitamin D.

**FIGURE 2:**
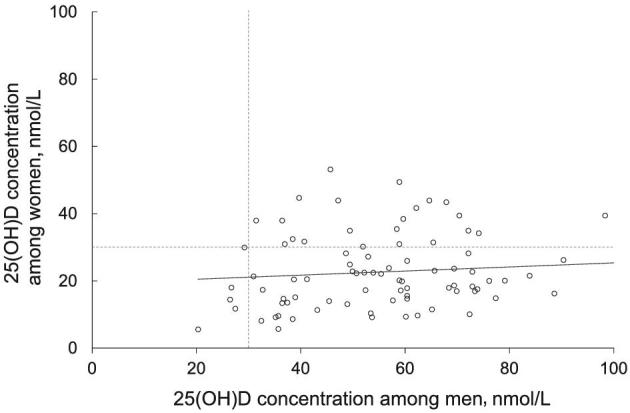
Association between plasma 25(OH)D concentrations of pregnant women (second trimester) and their husbands (*n* = 84 pairs). Among the married couples, there was no significant correlation between spouses’ vitamin D status (*r* = 0.1; *P* = 0.4). The dotted horizontal and vertical lines indicate the conventional cutoff for vitamin D deficiency (30 nmol/L) for women and men, respectively. 25(OH)D, 25-hydroxyvitamin D.

Pregnant women had significantly lower plasma 25(OH)D concentrations than their husbands (23 ± 11 compared with 55 ± 17 nmol/L; *P* < 0.001) (**Figure 1**). There was no significant correlation of 25(OH)D within spousal pairs (*r* = 0.1, *P* = 0.4) (**Figure 2**). Among women, 74% had 25(OH)D concentrations <30 nmol/L, whereas 6% of men were below this threshold. After adjusting for age and season of specimen collection, the 25(OH)D of pregnant women was 30 nmol/L lower (95% CI: −36, −25 nmol/L; *n* = 84) than that of men. The ICC was 0.09 (95% CI: 0.01, 0.6), indicating that 9% of total variation in 25(OH)D was attributable to pair-level variance. When each potential mediator was added to the regression model evaluating the relation between sex and 25(OH)D, the coefficient for sex remained essentially unchanged (**Table 2**), indicating no evidence of mediation by the included covariate. Among the hypothesized mediators, having an outdoor job had the greatest association with vitamin D status, but BMI and time spent outdoors were also related to vitamin D status (Table 2).

**TABLE 2:** Hypothesized mediators of the association between sex and 25(OH)D concentration among spousal pairs in Dhaka, Bangladesh^1^

Potential mediator	Difference in 25(OH)D concentration for a 1-unit or category change in listed potential mediator,^2^ nmol/L	Difference in 25(OH)D concentration between men and women,^3^ nmol/L
None		−30.5 (−36.3, −24.6)
BMI	−1.3 (−2.1, −0.4)	−30.1 (−36.4, −23.9)
Time spent outdoors per day,^4^ h	1.6 (0.4, 2.8)	−29.6 (−35.6, −23.6)
Engaged in an outdoor profession (vs. an indoors profession)	10.9 (2.2, 19.6)	−30.2 (−36.3, −24.1)
Sometimes or always used sunscreen (vs. rarely or never used)^5^	−2.4 (−12.8, 7.9)	−30.8 (−36.7, −24.8)

1 *n* = 84. Values are coefficients (95% CIs). 25(OH)D, 25-hydroxyvitamin D.

2 Based on a linear mixed-effects regression model to evaluate the relation between the potential mediator and plasma 25(OH)D concentrations. All models were adjusted for age and season of specimen collection. Coefficients and 95% CIs are for each potential mediator, in separate models, without sex as a covariate to test their separate effects on 25(OH)D.

3 Based on a linear mixed-effects regression model to evaluate the relation between sex and 25(OH)D concentrations with or without each potential mediator included. All models were adjusted for age and season of specimen collection. Coefficients and 95% CIs are for women (compared with men) when each potential mediator was included in the model.

4 Owing to missing data, *n* = 83 women.

5 Owing to missing data, *n* = 83 men and *n* = 83 women.

With respect to age, BMI, and sun exposure–related behaviors, women included in the comparison of pregnancy with postpartum were generally similar to the women included in the husband–wife comparisons (Table 1). The seasonal timing of specimen collection differed between the pregnancy and 6-mo-postpartum time points (Table 1).

Serum 25(OH)D concentrations were 26 ± 13 and 29 ± 12 nmol/L during pregnancy and at 6 mo postpartum, respectively (*P* = 0.03) (Figure 1). Adjusting for age, season of specimen collection, and BMI, 25(OH)D concentrations were similar during mid-pregnancy and at 6 mo postpartum (mean difference: −2.4 nmol/L; 95% CI: −5.3, 0.4 nmol/L; *P* = 0.1; *n* = 89 prenatal; *n* = 86 postpartum owing to missing BMI). The ICC was 0.43 (95% CI: 0.28, 0.61), indicating that 43% of the total variation in 25(OH)D was attributable to between-women variance, whereas 57% was attributable to between–time point (within-woman) variation including biological variability and imprecision in 25(OH)D measurement.

## Discussion

Comparison of the vitamin D status of a sample of pregnant women in Dhaka, Bangladesh with that of their husbands revealed a large gender disparity in 25(OH)D concentrations. Elimination of the gender disparity in vitamin D status could substantially reduce the prevalence of biochemical vitamin D deficiency [25(OH)D < 30 nmol/L] in women in this setting. The nonshared factors available for analysis in this cohort (BMI, times spent outdoors, involvement in an outdoor job, and sunscreen use) did not explain gender differences in 25(OH)D, and correlation of 25(OH)D within spousal pairs was weak. Overall, the present findings suggest that unmeasured or ubiquitous gender-specific environmental, social, or behavioral factors contribute to the relatively low 25(OH)D among women in Dhaka.

We did not find evidence to support our hypotheses that time spent outdoors or engagement in an outdoor profession explain the relation between gender and vitamin D status. Similarly, sunscreen usage is widely regarded to reduce cutaneous synthesis of vitamin D (6); yet, we did not find that sunscreen use was associated with 25(OH)D. We speculate that measurement error in self-reported measures of time spent outdoors, engagement in an outdoor profession, and sunscreen use may have attenuated the associations of these variables with vitamin D status. The reported sunscreen use by women was less frequent than expected, because a previous study of garment factory workers in Bangladesh showed that nearly 90% of women reported applying sunscreen on their faces and hands (11). We did not collect data on clothing practices of both men and women, or measure the amount of skin covered when outdoors, factors that would be expected to modify the amount of sun exposure independently of time spent outdoors. Bangladesh is a predominantly conservative Muslim society where many women cover their heads and limbs in public, and where sun avoidance may be more common among women than men. Clothing practices have been widely proposed as a contributing factor to vitamin D deficiency among women in the Middle East and South Asia (20–22). However, data that would enable comparisons of such factors across genders would be very challenging to obtain (23) (e.g., measures would have to quantify the proportion of skin exposed throughout the day, and take into account UVB penetration through various fabrics) and were not available in this trial cohort.

The weak correlation of 25(OH)D within spousal pairs indicated that shared (i.e., gender-nonspecific) household factors (e.g., living conditions, availability of vitamin D–rich foods in the home) are relatively unimportant determinants of vitamin D status in this setting. Although we did not compare the dietary vitamin D intakes of men and women, we assumed that vitamin D availability in the diet is a shared factor. Few foods in the Bangladeshi food supply are vitamin D–fortified (24) and supplement use is infrequent, so vitamin D intake is believed to be a minor contributor to vitamin D status in this setting. The prevalence of vitamin D supplement use by women in the general population in Bangladesh is unknown, but vitamin D supplementation is not currently promoted by a national public health strategy or program (25).

Previous husband–wife comparisons in Europe have found an absence of significant gender differences in 25(OH)D; however, similar to the present study, others have found that shared factors are unlikely to explain most of the variation in 25(OH)D in the population. For example, married Hindu Asian couples living in Britain were found to have very similar mean plasma 25(OH)D concentrations, even though the within-household correlation in 25(OH)D concentrations was low (26). Similarly, white married couples in France showed comparable 25(OH)D concentrations and weak within-pair correlations (27). In addition, in Saudi Arabia where traditional gender-specific roles predominate, a study of 50 husband–wife pairs found that the husbands had higher mean 25(OH)D concentrations than their wives (by 9 nmol/L; *P* < 0.001) (12). Therefore, the present findings are in agreement with previous studies with respect to the relative unimportance of shared/household factors, yet the observed husband–wife differential in 25(OH)D in Dhaka is larger than previously reported in other settings.

We also considered whether there may be a biological basis for the gender differential in 25(OH)D. Few studies have examined intrinsic biological effects of sex on vitamin D metabolism, and results are inconsistent. Mouse model studies showed that high concentrations of androgen found in males may act as inhibitors of cutaneous vitamin D synthesis (28, 29). For example, UV irradiation increased 25(OH)D concentrations more markedly in female mice and androgen-depleted male mice than in control male mice (28). However, another recent study showed that treating castrated mice with an androgen significantly increased blood 25(OH)D concentrations compared with those of vehicle-treated mice (30). Therefore, although the inconclusive biological role of sex on vitamin D status warrants further research, current available evidence does not suggest a mechanism by which women would have lower 25(OH)D due to biological differences alone. We specifically examined whether the pregnant state itself was the cause of the husband–wife differential in vitamin D status. However, we found that the change in 25(OH)D concentrations between mid-pregnancy and 6 mo postpartum was negligible. Direct effects of pregnancy on vitamin D metabolism include increases in the concentrations of vitamin D binding protein and calcitriol (31, 32); yet, similar to the present study, previous studies have reported a minimal influence of pregnancy on maternal 25(OH)D concentrations (32–34). The timing of data collection from the spousal pairs occurred only during the second trimester of pregnancy of the wives; however, moderate within-woman correlation (tracking) of 25(OH)D values from pregnancy to the postpartum period supported the notion that the 25(OH)D measurements in pregnant women were reasonable surrogates of the 25(OH)D in WRA in general. The observed within-woman variation from one time point to the next was expected and has been reported in other longitudinal studies of 25(OH)D tracking over time (34, 35).

This study was limited by a relatively small sample size and the lack of data related to numerous potential nonshared factors that may explain the gender differential in vitamin D status. We assumed that the availability of vitamin D–rich foods was low and would operate as a shared rather than a nonshared factor; however, as acknowledged, we did not measure dietary intake, so it is possible that intake of vitamin D–rich foods differed between men and women. Some research has indicated that Bangladeshi women report lower quality and lower amounts of preferred foods than do their husbands (36). However, a more recent study found that intrahousehold food allocation was not a common concern of women in Dhaka, and that food security was affected to a greater extent by factors that would typically be shared at the household level (e.g., rising costs of food and insufficient income) (37). We did not assess physical activity levels, which is a nonshared factor that was found to be an independent predictor of 25(OH)D in the aforementioned study of husband–wife pairs in Saudi Arabia (12).

In summary, husband–wife comparisons revealed an important gender disparity in vitamin D status in Dhaka, Bangladesh. Pregnant and lactating women in this study had a high prevalence of vitamin D deficiency that was not explained by household factors that were shared with their spouses or by a set of potential individual-level explanatory variables. We therefore speculate that ubiquitous societal practices and norms that limit women's UVB exposure [e.g., conservative dress (20–22)] likely constitute the major nonshared individual-level factors that explain the relatively low 25(OH)D among women in this setting. Future research and public health programs that aim to improve vitamin D status in Bangladesh should prioritize the characterization of such gender-specific risk factors as well as targeted strategies that will achieve higher 25(OH)D among pregnant and nonpregnant WRA.

## Notes

Supported by Bill & Melinda Gates Foundation grant OPP1066764.

Author disclosures: J-HJ, JK, EP, JS, ADG, AAM, and DER, no conflicts of interest.

The funders had no role in the design, implementation, analysis, and interpretation of the data.

Abbreviations used: ICC, intraclass correlation coefficient; icddr,b, International Centre for Diarrhoeal Disease Research, Bangladesh; MDIG, Maternal Vitamin D for Infant Growth; UVB, ultraviolet B; WRA, women of reproductive age; 25(OH)D, 25-hydroxyvitamin D.
